# TRmir: A Comprehensive Resource for Human Transcriptional Regulatory Information of MiRNAs

**DOI:** 10.3389/fgene.2022.808950

**Published:** 2022-02-04

**Authors:** Yu Gao, Chenchen Feng, Yuexin Zhang, Chao Song, Jiaxin Chen, Yanyu Li, Ling Wei, Fengcui Qian, Bo Ai, Yuejuan Liu, Jiang Zhu, Xiaojie Su, Chunquan Li, Qiuyu Wang

**Affiliations:** ^1^ School of Medical Informatics, Harbin Medical University, Daqing, China; ^2^ The First Affiliated Hospital, Department of Cardiology, Hengyang Medical School, University of South China, Daqing, China; ^3^ College of Medical Laboratory Science and Technology, Harbin Medical University, Daqing, China; ^4^ The First Affiliated Hospital, Institute of Cardiovascular Disease, Hengyang Medical School, University of South China, Hengyang, China; ^5^ School of Computer, University of South China, Hengyang, China; ^6^ The First Affiliated Hospital, Cardiovascular Lab of Big Data and Imaging Artificial Intelligence, Hengyang Medical School, University of South China, Hengyang, China; ^7^ Hunan Provincial Base for Scientific and Technological Innovation Cooperation, University of South China, Hengyang, China; ^8^ General Surgery Department, Beijing Friendship Hospital, Capital Medical University, Beijing, China; ^9^ Guangxi Key Laboratory of Diabetic Systems Medicine, Guilin Medical University, Guilin, China

**Keywords:** microRNA, super-enhancer/typical enhancer, chromatin accessibility, transcriptional regulation, genetics and epigenetics

## Abstract

MicroRNAs (miRNAs) are small non-coding RNAs, which play important roles in regulating various biological functions. Many available miRNA databases have provided a large number of valuable resources for miRNA investigation. However, not all existing databases provide comprehensive information regarding the transcriptional regulatory regions of miRNAs, especially typical enhancer, super-enhancer (SE), and chromatin accessibility regions. An increasing number of studies have shown that the transcriptional regulatory regions of miRNAs, as well as related single-nucleotide polymorphisms (SNPs) and transcription factors (TFs) have a strong influence on human diseases and biological processes. Here, we developed a comprehensive database for the human transcriptional regulation of miRNAs (TRmir), which is focused on providing a wealth of available resources regarding the transcriptional regulatory regions of miRNAs and annotating their potential roles in the regulation of miRNAs. TRmir contained a total of 5,754,414 typical enhancers/SEs and 1,733,966 chromatin accessibility regions associated with 1,684 human miRNAs. These regions were identified from over 900 human H3K27ac ChIP-seq, ATAC-seq, and DNase-seq samples. Furthermore, TRmir provided detailed (epi)genetic information about the transcriptional regulatory regions of miRNAs, including TFs, common SNPs, risk SNPs, linkage disequilibrium (LD) SNPs, expression quantitative trait loci (eQTLs), 3D chromatin interactions, and methylation sites, especially supporting the display of TF binding sites in the regulatory regions of over 7,000 TF ChIP-seq samples. In addition, TRmir integrated miRNA expression and related disease information, supporting extensive pathway analysis. TRmir is a powerful platform that offers comprehensive information about the transcriptional regulation of miRNAs for users and provides detailed annotations of regulatory regions. TRmir is free for academic users and can be accessed at http://bio.liclab.net/trmir/index.html.

## Introduction

MicroRNAs (miRNAs) are single-stranded small molecular RNAs, 21–23 bases in size produced by Dicer processing of single-stranded RNA hairpin loop precursors. As non-coding RNAs with regulatory functions, miRNA participate in various biological processes, including the development, organ formation, cell proliferation, differentiation, and fat metabolism (Inui et al., 2010; Li et al., 2018; Wang et al., 2018). For example, nuclear miR-122 can directly regulate survival via the regulation of miR-21 at the posttranscriptional level (Wang et al., 2018). In recent years, more abundant miRNA-related evidence has provided further insights into miRNAs and shown that some miRNAs were associated with various diseases such as cancers (Esquela-Kerscher and Slack, 2006; Shi et al., 2007; Sylvestre et al., 2007; Siva et al., 2009; Sun et al., 2009; Yang et al., 2013; Rupaimoole and Slack, 2017). Significant progress has been made in identifying miRNA targets and their association with cancers and diseases (Li et al., 2014; Georgakilas et al., 2016; Li et al., 2018; Palmieri et al., 2018; Wu et al., 2019). It is worth noting that miRNAs are often regulated by related super- or typical enhancers in addition to promoters (Duan et al., 2016; Suzuki et al., 2017; Sin-Chan et al., 2019; Ri et al., 2020). Typical enhancers, such as distal cis-regulatory DNA elements positively participate in the regulation of genes in a tissue-specific manner (Shlyueva et al., 2014). Super-enhancers (SEs) are emerging as clusters of enhancers that are densely occupied by master regulators and mediators and are thought to act as switches to determine the cell identity and fate (Hnisz et al., 2013; Whyte et al., 2013). From previous literature-based reviews, we found that typical enhancers/SEs could regulate the adjacent miRNAs (Matsuyama and Suzuki, 2019). For example, via integrated analysis of the potential connection between SEs and miRNAs, Young et al. found that SEs were related to many miRNAs and master transcription factors (TFs), and they reported on the relationship between SE-miRNAs and cancers (Suzuki et al., 2017). The transcription of miR-146a and miR-155, driven by SEs, in turn downregulates both *in vitro* and *in vivo* canonical inflammatory genes expression by targeting inflammatory mediators (Duan et al., 2016). Ri et al. found that the overexpression of miR-1301 induced by the Klf6 SE could lead to significant inhibition of proliferation in human hepatoma HepG2 cells (Ri et al., 2020). In addition, recent studies have suggested that single-nucleotide polymorphisms (SNPs) within enhancers could affect TF binding sites in the regulation of diseases (Izzi et al., 2016; Liu et al., 2017). A possible role for the epigenetic regulation in regulating miRNA expression has also been reported by some researchers (Ramassone et al., 2018; Yao et al., 2019). Epigenetic regulation includes DNA methylation and chromatin/histone modifications, all of which can participate in regulating miRNA expression. Some studies have shown that over 100 miRNAs were epigenetically regulated in different cancers, and the methylation frequency of human miRNA genes appeared to be much higher than that of protein-coding genes (Weber et al., 2007; Kunej et al., 2011). Consistent with these findings, researchers have found that miRNA genes frequently overlapped not only the cancer-associated genomic regions but also the CpG islands (Calin et al., 2004; Morales et al., 2017). One study showed that epigenetic modifications within mir290 enhancers dynamically altered switching, resulting in cell-to-cell heterogeneity (Song et al., 2019). Zhao et al. highlighted how chromatin states directed miRNA-mediated network motifs by integrating the epigenome and regulatome (Zhao et al., 2016). All of this evidence emphasizes the importance of integrating and calculating miRNA-related transcription regions and the regulation of genes within these regions (epi).

Many miRNA databases have been built, such as HMDD (Li et al., 2014), IMOTA (Palmieri et al., 2018), DIANA-miRGen v3.0 (Georgakilas et al., 2016), piRTarBase (Wu et al., 2019), DIANA-TarBase (Vlachos et al., 2015), mirDIP (Tokar et al., 2018), TFmiR (Hamed et al., 2015), mirTrans (Hua et al., 2018), and TransmiR v2.0 (Tong et al., 2019). However, these existing databases only support a small amount of genetic data and annotation information within miRNA promoter regions. They ignore the importance of information within the transcriptional regulatory regions (especially the typical enhancer/SE/chromatin accessibility regions of miRNAs). With the development of next-generation sequencing technology, we can obtain more H3K27ac and ChIP-seq data, which can be used to identify typical enhancers, SEs, and more ATAC-seq data, and this can be used to identify chromatin accessibility regions. Consequently, there is an urgent need to integrate and process existing resources to establish a database that contains more comprehensive information about the transcriptional regulation of miRNAs.

Based on the earlier analysis, we established a database which could provide more comprehensive transcriptional regulatory information and annotation information for miRNAs. First, we collected as many samples as possible and used process frameworks to identify miRNA regulatory regions from more than 900 ATAC-seq, H3K27ac ChIP-seq, and DNase-seq samples. Furthermore, in order to enable researchers to further understand the transcriptional regulatory mechanisms of miRNAs, we provided more detailed annotation information about the transcriptional regulatory regions of miRNAs, such as TFs collected by ChIP-seq or predicted by FIMO (Grant et al., 2011) and methylation sites from multiple sources and other regions. In addition, TRmir provided additional information about miRNAs including miRNA-related diseases, extensive pathway analysis, and miRNA expression. It can be seen from Table 1 that our database was far superior to other databases in both the number of transcriptional regulatory entries and annotation information. In conclusion, TRmir was a human miRNA transcriptional regulation database, which integrated data storage, friendly interface query, detailed annotation, online analysis, and other functions.

**TABLE 1:** Summary of the contents of TRmir and other comparable databases.

Database	miRNAs	miRNA TSSs	TF-miRNA regulations	TE-miRNA regulations^1^	SE-miRNA regulations^2^	Chromatin accessibility- miRNA regulations	Common SNPs	eQTLs	Risk SNPs	Methylation sites	3D chromatin interactions
TRmir (our database)	1,684	12,549	34,077,855	5,455,844	298,570	1,733,966	38,063,729	2,886,113	264,514	198,468,712 (Sites)	29,137,183 (interactions)
										161 (samples)	292 (samples)
										109 (sample types)	145 (sample types)
mirTrans (2017)	1,513	35,259	2,340,406	—	—	—	—	—	—	—	—
TransmiR (2019)	100	—	735	—	—	—	—	—	—	—	—
EnhancerDB (2019)	1,726	Unkown	4,039,558	17,059	—	—	11,381,519	119,938	—	—	—
DIANA-miRGen(2016)	428	276	Unkown	—	—	—	—	—	—	—	—
ChIPBase (2016)	Unkown	Unkown	273,761	—	—	—	—	—	—	—	—
TsmiR (2014)	116	—	2,347	—	—	—	—	—	—	—	—
CircuitsDB (2013)	180	—	115 TFs to 180 miRNAs	—	—	—	—	—	—	—	—
miRT (2012)	588	670	—	—	—	—	—	—	—	—	—
miRDB (2020)	2,656	Unkown	—	—	—	—	—	—	—	—	—
mirBase (2019)	1,918	—	—	—	—	—	—	—	—	—	—
mirWalk (2020)	2,656	Unkown	—	—	—	—	—	—	—	—	—

1 TE-miRNA regulations: the regulatory relationship between typical enhancers and miRNAs.

2 SE-miRNA regulations: the regulatory relationship between super-enhancers and miRNAs.

## Database Content and Methods

### Identification of Transcription Regulatory Regions

Because the primary miRNA transcription product (pre-miRNA) is cleaved into a precursor miRNA by RNase Drosha in the nucleus (Hamed et al., 2015), the mechanisms underlying miRNA transcription are unclear due to the lack of experimental methods for detecting miRNA transcription start sites (TSSs) with high resolution. Thanks to the recent development of high-throughput deep sequencing techniques, the identification of miRNA TSSs has become more accurate (Consortium et al., 2014). Aiming to more accurately identify miRNA promoter regions, we integrated TSSs from miRbase (Griffiths-Jones et al., 2008) and microTSS, which can provide highly accurate TSSs for miRNAs (Georgakilas et al., 2014). Importantly, we applied microTSS as the first algorithm on sequenced RNA-, ChIP-, and DNase-Seq data. Finally, we obtained 12,549 TSSs for 1,684 miRNAs. We obtained the promoter region by extending the upstream and downstream sequences from the transcription start site (e.g., 5 kb/1 kb). Moreover, we integrated the details of miRNAs by referring to miRBase (Griffiths-Jones et al., 2008) and DIANA-miRGen v3.0 databases (Georgakilas et al., 2016). For the sake of version uniformity, we used the liftOver tool of UCSC (Fujita et al., 2011) to convert the genomic locations of miRNAs.

**FIGURE 1:**
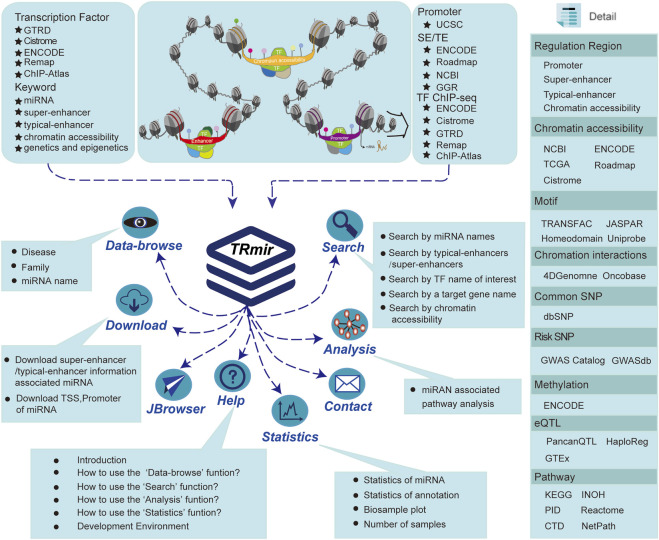
Database introduction. Our database provides the most abundant information about human miRNA regulation. In addition to providing four regulatory regions, we also collected a large quantity of raw data from a variety of resources in order to provide more comprehensive regulation and annotation information. TRmir is a database platform integrating storage, visualization, analysis, and friendly query.

We collected H3K27ac, ChIP-seq, and ATAC-seq data of various samples from public databases. Following a unified and standardized analysis process, we identified the DNA regulatory elements of all samples, including SEs, enhancers, and chromatin accessibility regions. Aiming to identify typical enhancer/SE regions, we collected H3K27ac ChIP-seq sequencing data from hundreds of different tissues/cells in multiple databases such as NCBI GEO/SRA (Barrett et al., 2011), Roadmap (Bernstein et al., 2010), ENCODE (Consortium, 2012), and GGR (Figure 1; Supplementary Table S1) (Lovén et al., 2013). We used Bowtie (Langmead et al., 2009; Fujita et al., 2011; Hnisz et al., 2013) to align the reads to the reference genome. Next, we used MACS (v1.4.2) (Zhang et al., 2008) with the command “macs14 -p 1e-9 -w -S --keep-dup = auto–wig--single-profile --space = 50” to further identify the enrichment information of H3K27ac, including peak position information and credibility. Finally, we used ROSE (Lovén et al., 2013) to identify SEs. In the recognition process, we stitched together the enhancers with a range of 12.5 kb and then sorted them according to the signal strength. We distinguished the threshold between SEs and enhancers based on the signal value obtained from the tangent point of the tangent with a slope of 1. DNase-seq and ATAC-seq (Meyer and Liu, 2014) as the more popular sequencing technologies were used for the identification of chromatin accessibility regions. For DNase-seq data, we obtained 290 DNase-seq samples of various cells/tissues from ENCODE (Consortium, 2012), Roadmap (Bernstein et al., 2010), and Cistrome (Mei et al., 2017). ATAC-seq data were a valuable resource for the systematic investigation of gene regulatory processes and supplied a wealth of information on the susceptibility, mechanisms, prognosis, and potential therapeutic strategies of diverse cancer types (Meyer and Liu, 2014). ATAC-seq is a sequencing method that uses Tn5 transposase to capture open regions in nuclear genomic DNA. We manually collected 128 ATAC-seq samples bed files from publicly available human ATAC-seq datasets in three resources including Cistrome (Mei et al., 2017), NCBI (Barrett et al., 2011), and TCGA (Corces et al., 2018) (Supplementary Table S2). The Python script GeneMapper.py from ROSE was used to predict the related regions using three different strategies. It is worth noting that these regions have been shown to loop with neighboring genes (Suzuki et al., 2017). All pipelines were written using the RefSeq (GRCh37/hg19) human gene annotations. Finally, we obtained 5,754,414 typical enhancers/SEs and 1,733,966 chromatin accessibility regions associated with miRNAs.

### Annotation of Related Regulatory Regions

In order to further explore the function of miRNAs, we provided detailed annotation information for each transcriptional regulatory region of miRNAs. First, we obtained more than 7,000 ChIP-seq datasets of 952 TFs from ENCODE (Consortium, 2012), Cistrome (Mei et al., 2017), Remap (Chèneby et al., 2018), ChIP-Atlas, and GTRD (Yevshin et al., 2017). Each database carried out strict quality control on ChIP-seq data. And then the liftOver tool of UCSC was used to convert these peak datasets to the latest genome assemblies, and regions that failed to transfer were discarded. We obtained over 3,000 DNA-binding motifs for ∼700 TFs, which were collected from JASPAR CORE 2014 vertebrates (Mathelier et al., 2014), Jolma 2013 (Jolma et al., 2013), homeodomains (Berger et al., 2008), UniPROBE (Robasky and Bulyk, 2011), and Wei 2010 (Wei et al., 2010). At the same time, the FIMO (Grant et al., 2011) with the command “fimo -verbosity 1 —thresh 1e-6” from the MEME suite (Bailey et al., 2009) was used to scan the sequences for inferred motifs. In addition, we downloaded 450 K methylation array data and whole-genome shotgun bisulfite sequencing data from ENCODE (Consortium, 2012). Finally, we obtained 198,468,712 methylation sites in total. We used beta values as the metric to measure the level of methylation. Furthermore, we used BEDTools (v2.25.0) with the command “bedtools intersect -a a. bed -b b.bed” and set all the allowed overlap fractions from BEDtools intersect defaults to 1 bp (Quinlan and Hall, 2010) in order to identify the methylation sites, which overlapped the transcriptional regulatory regions of miRNAs.

Second, we obtained common SNPs from dbSNP (Sherry et al., 2001) and calculated the SNPs with a minimum allele frequency over 0.05 by using VCFTools (v0.1.13) (Danecek et al., 2011). Finally, we obtained 38,063,729 common SNPs. At the same time, we calculated LD SNPs (*r* ^2^ = 0.8) for the five superpopulations, which contained South Asian, European, East Asian, Ad Mixed American, and African populations by using plink (v1.9) (Purcell et al., 2007). In addition, we collected over 260,000 risk SNPs from the GWAS catalog (Welter et al., 2014) and GWASdb v2.0 (Eicher et al., 2015). We also obtained over 2,886,000 human eQTLs and 31,080,000 eQTL-gene pairs from GTEx v5.0 (Carithers and Moore, 2015), HaploReg (Ward and Kellis, 2012), and PancanQTL (Gong et al., 2018). Finally, in order to validate the regulatory relationships predicted by our database, we directly downloaded 179 samples of Hi-C and ChIA-PET in BED file format from 4DGenome (Teng et al., 2016) and OncoBase (Li et al., 2019) (Supplementary Table S3).

### Functional Annotations of miRNAs

Aiming to facilitate researchers who wish to perform a systematic investigation of the transcriptional regulation of miRNAs, we provided additional miRNA information, including the expression of miRNAs from multiple cancers, miRNA-related diseases, and pathway analysis. In order to assist users in obtaining the expression value of miRNAs in different cancers, we downloaded the matrix expression data of 33 types of cancers and pan-cancers, respectively (Corces et al., 2018). The miRNA target gene data were extracted from miRTarBase (Hsu et al., 2011) and were subsequently manually curated based on a high-accuracy text-mining system and aims to accumulate experimentally validated miRNA–target interactions (MTIs). We collected a large quantity of miRNA–disease–related information from HMDD v3.0 (Li et a0l., 2014), including the associated disease name, the confirmed literature PubMed ID, and the description.

### Identification of miRNA Upstream Pathways

In order to better understand the regulation mechanism of miRNA, we provided analysis functions for pathways that regulated miRNAs. Therefore, we collected 2,880 pathways and related information from our previous work ComPAT (Su et al., 2021). When users submit an miRNA, we first identify the relevant TFs that regulate the miRNA. Then, we use those TFs for pathway enrichment and obtain significantly enriched pathway information related to the miRNA by using the hypergeometric test (Quinlan and Hall, 2010; Li et al., 2013; Feng et al., 2016). We calculated the *p*-value for significant enrichment using the following formula:

$$\text{P}=1-\sum_{\text{i}=0}^{\text{x}-1}\frac{\left(\begin{array}{l} k\\ i \end{array}\right)\left(\begin{array}{l} n-k\\ s-i \end{array}\right)}{\left(\begin{array}{l} n\\ s \end{array}\right)}.$$ (1)

We then used the phyper function to realize the calculation of Eq. 1 using x as the number of genes involved in the pathway, s as the number of genes of interest, n as the total number of genes in the pathway, and k as the number of intersections between the genes in the pathway and the genes input by the user.

## Results

### Introduction to Database Usage

**FIGURE 2:**
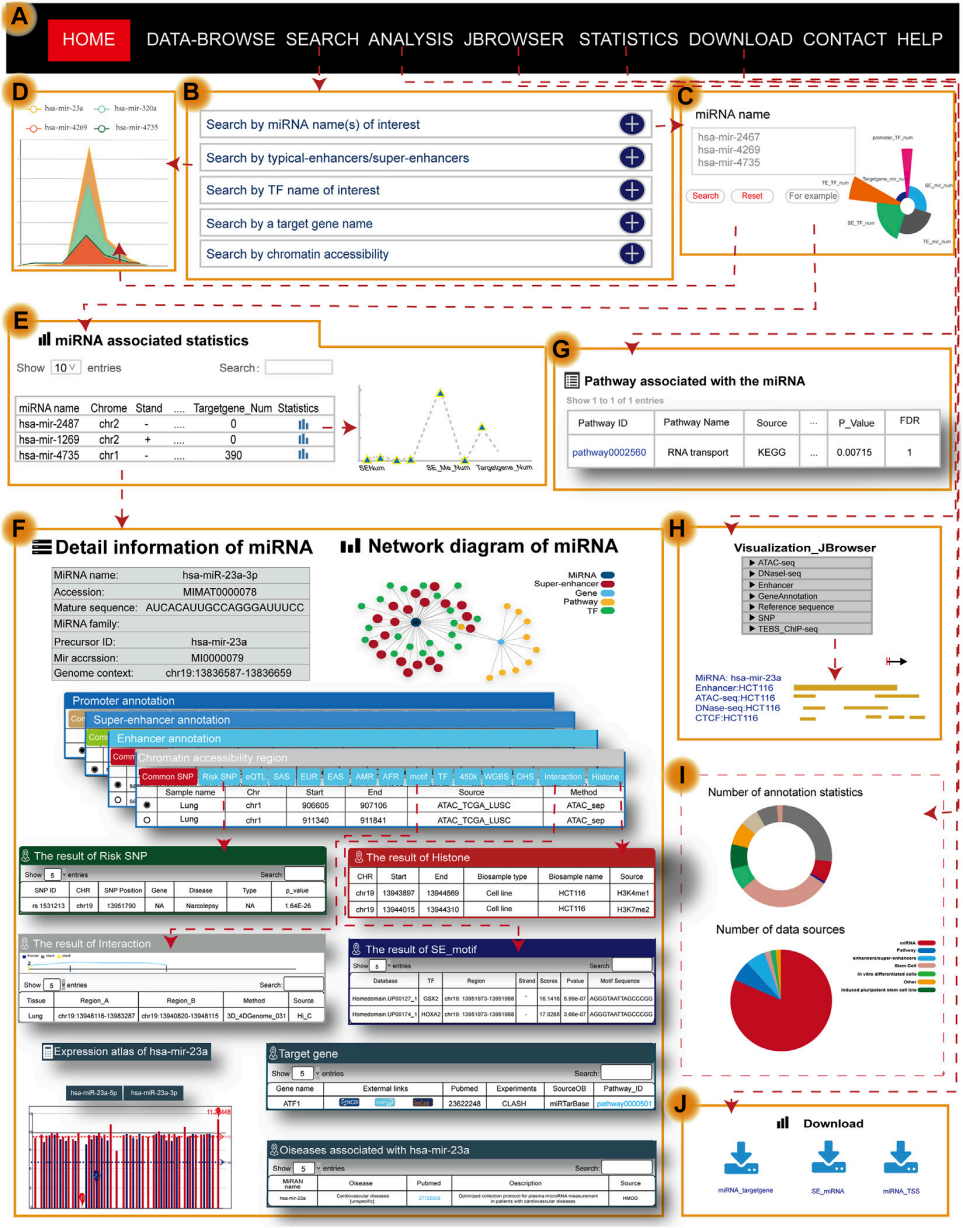
Main functions and usage of TRmir. **(A)** The navigation bar of TRmir. **(B)** Five query methods: “Search by miRNA name(s) of interest,” “Search by typical enhancer/super-enhancer,” “Search by TF name of interest,” “Search by a target gene name,” and “Search by chromatin accessibility.” **(C)** Advanced search is initiated by inputting the miRNA name(s) of interest. **(D)** Figure display of statistics associated with the miRNAs. **(E)** The table displays the statistics for the detailed (epi)genetic information of different regulatory regions. **(F)** Detailed information about the miRNA: general information about the miRNAs and target genes, the expression of each miRNA, and mean values for each sample, diseases associated with the miRNA and detailed genetic annotations. **(G)** Pathway analysis: detailed information from the pathway analysis. **(H)** Visualization of JBrowser. **(I)** Statistics of TRmir. **(J)** Download page of TRmir.

Users can search for the transcriptional regulatory information of miRNAs by five approaches, including “search by miRNA name(s) of interest,” “search by typical enhancer/super-enhancer” [input genomic position, sample], “search by TF name of interest,” “search by a target gene name,” and “search by chromatin accessibility” [input genomic position, sample] (Figure 2A–C). Users can obtain brief summary information of search results in a table (Figure 2E). The statistics in the table describe the genetic annotation of the three regions (Figure 2D). If users want to obtain more information about miRNA, they can click the “miRNA name” (Figure 2F). Users will then quickly see the general information about miRNA including the miRNA name, accession, mature sequence, miRNA family, precursor ID, and genome context. In addition to the general details, the network diagram intuitively and vividly shows not only the regulatory relationships among miRNAs (dark blue nodes), TFs (green nodes), and SEs (red nodes) but also the pathway name (yellow nodes) and target gene (light blue nodes) associated with miRNA (Figure 2F). At the same time, TRmir can provide information about the different regulatory regions of miRNA including, I: promoter (genomic position, TSS, and cell); II: SE/typical enhancer (enhancer ID, genomic position, element, size, rank, ChIP density, and is super, sample ID); and III: chromatin accessibility (genomic position, sample name, and source). We also provided more detailed annotation information for the three regulatory regions mentioned before including common SNPs, risk SNPs, eQTLs, TFs, and methylation sites (450 K array, whole-genome shotgun bisulfite sequencing), histone modifications, and 3D chromatin interactions (Figure 2F). For example, when users click the “Risk SNP” button within the SE region, TRmir can provide SNP ID, SNP position, gene, disease, type, and *p*-value for risk SNPs (Figure 2F). In the “Histone” module of the enhancer region, users can obtain the CHR, start, end, biosample type, biosample name, and source for the histone associated with the enhancer region (Figure 2F). When users input hsa-mir-23a and click the “motif” button within the SE region, TRmir can show the motif sequence, the source of DNA-binding motifs, TF name, and TF region (Figure 2F). As an example, when users input hsa-mir-23a (sample type: tissue, tissue: lung, sample name: lung; Figure 2F), they can find that the relationship between miRNA and the promoter was validated by chromatin interaction data from the “Interaction” module. Importantly, genome-wide identification, detailed annotation, and regulatory relationships of different regulatory regions are cell type-specific. Therefore, if users want to see different sample settings on the details page, they can customize the filter by clicking the sample option located in the middle of the page (Figure 2F). TRmir also provides additional information including miRNA expression, associated diseases, and target genes.

### Online Analysis Tools

To help users interactively analyze and understand the roles of miRNAs and their regulatory mechanisms in humans, TRmir provides miRNA pathway analysis. TRmir can identify TFs, which are downstream from the pathways binding to the related regions of miRNA. When users search the database by an miRNA name, TRmir can return those significantly enriched pathways using the hypergeometric test. The pathway analysis of miRNAs should greatly facilitate the study of regulatory mechanisms. The results table returns the enriched pathways and the related detailed information list. From the list, the user can obtain the pathway ID, pathway name, source, annotated gene of enrichment, annotated gene number, FDR, and *p*-value of the enrichment score (Figure 2G). If users want to obtain more information about the pathway, they can click the “Pathway ID” to jump to the detailed information page.

### User-Friendly Data Visualization and Personalized Genome Browser

To allow users to quickly browse data, we used bootstrap technology to develop a friendly interface for users to browse. Furthermore, users can automatically select items to browse by selecting “Family” and “Disease” from the navigation bar on the left. Users can easily click the “miRNA name” to further understand the transcriptional regulatory information for miRNA. For better visualization of information in the genome, we used a plugins named JBrowse (Figure 2H), which is compatible with browsers and built on JavaScript and HTML5 (Buels et al., 2016). Furthermore, TRmir also provides graphic visualization of chromatin interactions, quantitative statistics of annotation information within regulatory regions, and especially supports the relationship between TFs and miRNAs.

### Data Download and Statistics

Users can quickly download the file of interest by clicking the corresponding icon links (Figure 2J). The “Statistics” page on the website of TRmir provides a detailed statistical table of the miRNA transcriptional regulatory regions and annotation information (Figure 2I).

### Website Design and Development

We used MYSQL 5.7.17 for storage of the website, a lightweight database management system run on a Linux-based Web server. The website was built based on CSS3, PHP 8.0, and HTML5 frameworks, D3 (https://d3js.org), ECharts, and Highcharts. Aiming to facilitate browsing by users, we used Bootstrap v3.3.7 and JQuery v2.1.1 to design a friendly visual interface. At the same time, JBrowse was built for the visualization of data.

### Case Study

**FIGURE 3:**
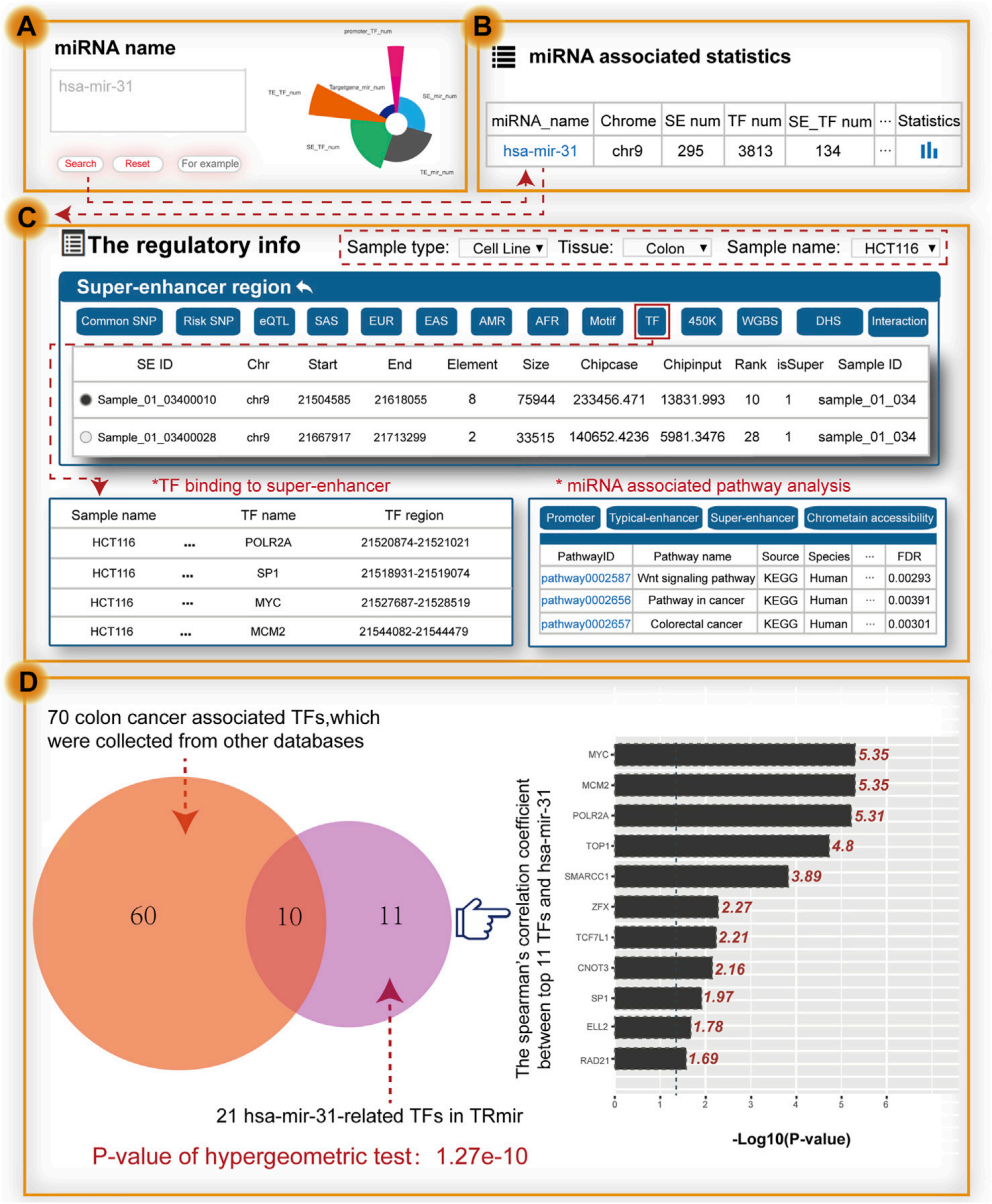
Main functions and usage of TRmir. Relevant validation results were obtained by inputting hsa-mir-31. **(A)** Search by miRNA. **(B)** Brief statistics on genetic annotation of hsa-mir-31. **(C)** From the perspective of the SE region shown on the details page for hsa-mir-31, we can obtain detailed information about pathway analysis, and TFs enriched in the regulatory regions. **(D)** Analysis of hsa-mir-31–related TFs. These related TFs are enriched in the related regulatory regions. The right panel shows the calculation results for Spearman’s coefficient (*p*-value = 0.05, the −logP-value cutoff value is 1.301).

To further validate the value of using TRmir, we took the small non-coding RNA hsa-mir-31 as an example, which is associated with colon cancer (Figure 3A). To validate the search results of our database, we collected experimental data from high quality journal literature (Suzuki et al., 2017). When users search the miRNA name by inputting hsa-mir-31, the results page first shows the statistics of hsa-mir-31 (Figure 3B). Notably, detailed information about hsa-mir-31 can be obtained by clicking the “miRNA name” to view the miRNA-enhancer-gene network and detailed annotation information within transcriptional regulatory regions in HCT116 cells (sample type: cell line, tissue: colon, sample name: HCT116; Figure 3C). From the “super-enhancer region” of TRmir, we found 22 SEs associated with hsa-mir-31 and 14 out of 22 SEs completely overlapped with the results of a study by Richard A Young (Suzuki et al., 2017). In the “super-enhancer region,” we found the sample_01_03400028 in the SE of hsa-mir-31, which was reported to show that the changes of SEs affect the progression of cancer (Suzuki et al., 2017). Moreover, hsa-mir-31 with gain of a SE in colon cancer cells displayed an increased prognostic value relative to miRNAs with SE loss (Suzuki et al., 2017). To summarize, our database on the transcriptional regulation of miRNAs provided a new insights for deeply understanding the transcriptional regulatory mechanism of miRNAs.

The relationship between SEs and TFs is important for the study of regulatory mechanisms. When we click the button named “TF,” TRmir shows hsa-mir-31-associated TF binding sites within the regulatory regions. We found that these 21 hsa-mir-31–related TFs were highly consistent with colon cancer–related TFs, such as APC, ARID1A, MCM2, MYC, TCF3, TP53, SP1, and TOP1, which were collected from DisGeNET (Piñero et al., 2017) and PTMD (Xu et al., 2018). For example, oncogenic MYC expression has been reported to be promoted by WNT signaling and AHCTF1 through SE-mediated gene gating and to increase the rate of colon cancer cell proliferation (Perdikopanis et al., 2021). We also found that TF ELL2, not reported in existing studies, was associated with colon cancer. We used the expression data of colon adenocarcinoma (COAD) from TCGA to calculate Spearman’s correlation coefficient, with the aim of further exploring the relationship between the expression of 21 TFs and hsa-mir-31 (Figure 3D). According to the results of the calculations, most of the TFs aforementioned were closely related. Furthermore, we used the TFs to identify hsa-mir-31-associated pathways in TRmir for pathway downstream analysis. From the results of the analysis, we can see that three pathways including the “Wnt signaling pathway” and the “colorectal cancer pathway” were significantly enriched. We have provided this example to help users understand how to use TRmir. The interaction of TFs and hsa-mir-31 associated with colorectal cancer indicated the utility of our database.

Similarly, as another example, we used the miRNA named “hsa-let-7b” as the input for “Search by miRNA name(s) of intersect.” hsa-let-7b was significantly enriched in human pericardial fluid, and enhanced expression of hsa-let-7b has been experimentally linked to cardiovascular disease (Beltrami et al., 2017). On the results page, users first obtained the “Detail information of miRNA.” After clicking the “miRNA name,” TRmir provided the network diagram of hsa-let-7b and regulatory information about hsa-let-7b. When we set the sample name as the heart left ventricle (sample type: tissue, tissue: heart left ventricle, sample name: heart left ventricle), we could find an SE named the “sample_00_01400330” from the “Super-enhancer region.” When users clicked the “TF” button in the “Super-enhancer region,” we found that GATA4 occupied the hsa-let-7b related SE region. GATA4 played an important role in heart development, cardiomyocytes, and cardiovascular disease, and has been extensively studied (Heikinheimo et al., 1994; Molkentin et al., 1997). For example, Ang et al. provided the regulatory landscape regarding GATA4 in human cardiac development and function. GATA4 widely co-occupied the cardiac SEs which cause dysregulation of genes, leading to cellular dysfunction in human cardiomyocytes (Ang et al., 2016). More importantly, in the section “Diseases associated with hsa-let-7b,” hsa-let-7b was associated with cardiovascular disease. These results demonstrated the availability and biological value of using TRmir for miRNA research (Supplementary Figure S1).

## Discussion

miRNAs are important small non-coding RNAs, which play important roles in the transcriptional regulation of biological processes. The regulation of miRNAs is associated with various regulatory regions and not just the promoters. With the development of second-generation sequencing, additional H3k27ac ChIP-seq and ATAC-seq data have become available. It is important to establish a database, which contains a comprehensive listing of transcriptional regulatory regions and extensive genetic annotations. In recent years, many popular databases including mirTrans (Hua et al., 2018), TransmiR (Tong et al., 2019), miRTarBase (Hsu et al., 2011), HMDD (Li et al., 2014), DIANA-TarBase (Vlachos et al., 2015), and DIANA-miRGen (Georgakilas et al., 2016) have been published to aid researchers in exploring the valuable resources pertaining to miRNAs. For example, miRTarBase (Chou et al., 2018) and DIANA-TarBase (Karagkouni et al., 2018) are miRNA target gene databases supported by experimental data. In addition, miRDB (Chen and Wang, 2020) and mirWalk (Sticht et al., 2018) are both online databases for miRNA target prediction with machine learning methods. The miRBase (Griffiths-Jones et al., 2006) database is a searchable database of published miRNA sequences and annotations. To improve the understanding of miRNAs some databases have been established, which describe the relationship between miRNAs and diseases. HMDD (Huang et al., 2019), as one of the more popular ones, is a manually collected miRNA and a disease-related database. However, compared to the abundance of miRNA target databases and miRNA–disease databases, resources describing TF-miRNA regulatory relationships are limited. Therefore, additional databases about miRNA transcription have been constructed to provide information about the TF-miRNA regulation, such as DIANA-miRGen v3.0 (Perdikopanis et al., 2021) and CircuitsDB (Friard et al., 2010). mirTrans (Hua et al., 2018) and TransmiR v2.0 (Tong et al., 2019) are both resources for the transcriptional regulation of miRNAs in human cell lines. In particular, TransmiR, which manually collected 2,852 TF-miRNA entries from 1,045 publications, has been upgraded to version 2.0. Until now, only one database named EnhancerDB (Kang et al., 2019) has provided a small amount of data on regulatory relationships between enhancers and miRNAs, but it is not very comprehensive (Table 1). All of the databases aforementioned have made great contributions to miRNA studies, but these studies and databases have only emphasized the importance of small genetic annotations of miRNAs (Li et al., 2014; Zhao et al., 2016; Song et al., 2019). None of these resources were developed to provide the transcriptional regulatory regions for miRNAs and genetic annotations were also ignored. However, studies have now increasingly indicated that important factors affecting the miRNA transcriptional regulation are not only associated with promoter regions but also with other regions such as chromatin accessibility regions and super- or typical enhancers, which play an important role in transcriptional processes of miRNAs (Duan et al., 2016; Suzuki et al., 2017; Sin-Chan et al., 2019; Ri et al., 2020). Therefore, we developed the TRmir database, which can provide more comprehensive resources for understanding the regulatory mechanisms of miRNAs. Compared with existing databases, TRmir allows researchers to easily obtain information about different regulatory regions. From Table 1, we can find the major differences between TRmir and other databases, especially in terms of the number of some terms, such as miRNAs, enhancers, TSS, and open chromatin regions. Furthermore, it provides the most abundant annotation information for the above regulatory regions. We compared the regulatory relationship between TF and miRNA in TRmir with the experimentally validated regulatory relationship in Transmir. We found that most of the TF-miRNA regulatory relationships in TRmir significantly overlapped with those in TransmiR. For example, GATA1-miRNA regulations in TRmir are significantly enriched in GATA1-miRNA regulations from TransmiR (hypergeometric test; *p*-value = 2.95e-14). The *p*-value of the hypergeometric test for NFYB-miRNA is 1.26e-78 (Supplementary Figure S2; Supplementary Table S4). The result indicated that the TF-miRNA regulations in our database are reliable and robust. Finally, in addition to miRNA-related expression and target genes, pathway analysis was also provided.

Our motivation to build this database comes from the huge demand of geneticists and biologists to understand the regulatory mechanism of miRNAs. The current version of TRmir stores the most abundant comprehensive transcriptional regulatory information and (epi)genetic annotations of human miRNAs. We believe our database will be useful, but it does have some limitations. For example, a ranking metric would be useful for the user because there is likely to be a daunting amount of information coming from most searches. The implementation of a score may help users focus on specific miRNAs. Therefore, in future versions, we plan to provide a ranking metric such as a score to combine expression, TF hits, accessibility, SE annotation, motif presence, interaction, and other data.

## Conclusion

TRmir aims to provide a resource with the most informative transcriptional regulatory regions for miRNAs, and detailed annotation information within the regions. In order to facilitate deeper understanding of the transcriptional regulation of miRNAs, we have provided a large amount of annotation information located in the regulatory regions. In particular, we have provided the TFs that are obtained by two methods: TFs supported by ChIP-seq technology and TFs predicted by motif. In addition, we also provide information regarding methylation sites, one based on 450 K array data and the other based on whole-genome shotgun bisulfite sequencing. At the same time, TRmir integrates miRNA expression and related disease information and supports extensive pathway analysis. TRmir has a friendly interface to provide a good user experience and is convenient for users to query and browse, especially as it provides a comprehensive transcriptional regulation database of miRNAs for users with detailed regulatory annotation about these regions.

## Data Availability

The datasets presented in this study can be found in online repositories. The names of the repository/repositories and accession number(s) can be found in the article/Supplementary Material.
