# Supplementary material for: TRmir: A Comprehensive Resource for Human Transcriptional Regulatory Information of MiRNAs
Source: Front Genet. 2022 Feb 4;13:808950. doi: 10.3389/fgene.2022.808950 (PMC8854293; doi:10.3389/fgene.2022.808950)
Supplement: Supplementary file 2 [file Table1.PDF]

Supplementary Table 1. The statistics of datasets in TRmir.

Element (datasets)	Source
Enhancers/ Super-enhancers (H3K27ac ChIP-seq)	ENCODE, NCBI, Roadmap, GGR
Chromatin accessibility (ATAC-seq, DNase-seq)	Cistrome, TCGA, ENCODE, Roadmap, NCBI
TF (ChIP-seq)	Cistrome, ENCODE, Remap, GTRD, ChIP-Atlas
Methylation (450K, WGBS)	ENCODE
Common SNP	dbSNP
eQTL	PancanQTL, HaploReg, GTEx
Risk SNP	GWAS Catalog, GWASdb
Chromatin interactions (e.g., ChIA-PET, 3C, 4C, 5C and Hi-C)	4DGenome, Oncobase, The 3D Genome Browser, NCBI
Expression	TCGA (https://tcga-data.nci.nih.gov/tcga)
Diseases	HMDD v3.0
Experimentally confirmed target gene	miRTarBase (release 6.0)
miRNA locations	microTSS(Georgakilas.,NC, 2014)
