# Supplementary figures and images for: TRmir: A Comprehensive Resource for Human Transcriptional Regulatory Information of MiRNAs
Source: Front Genet. 2022 Feb 4;13:808950. doi: 10.3389/fgene.2022.808950 (PMC8854293; doi:10.3389/fgene.2022.808950)

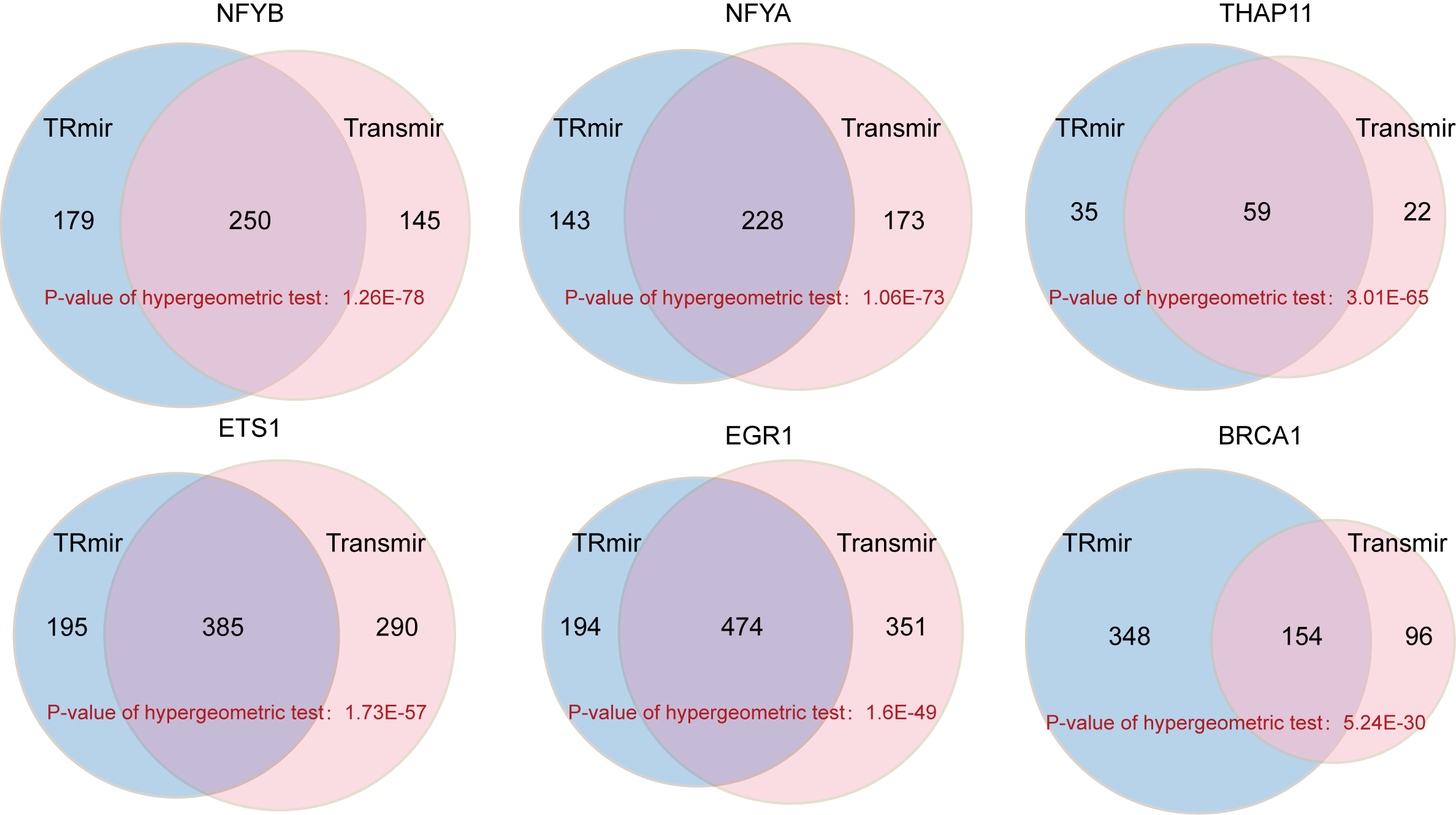

Supplement: Supplementary file 5 [file Image2.TIF]

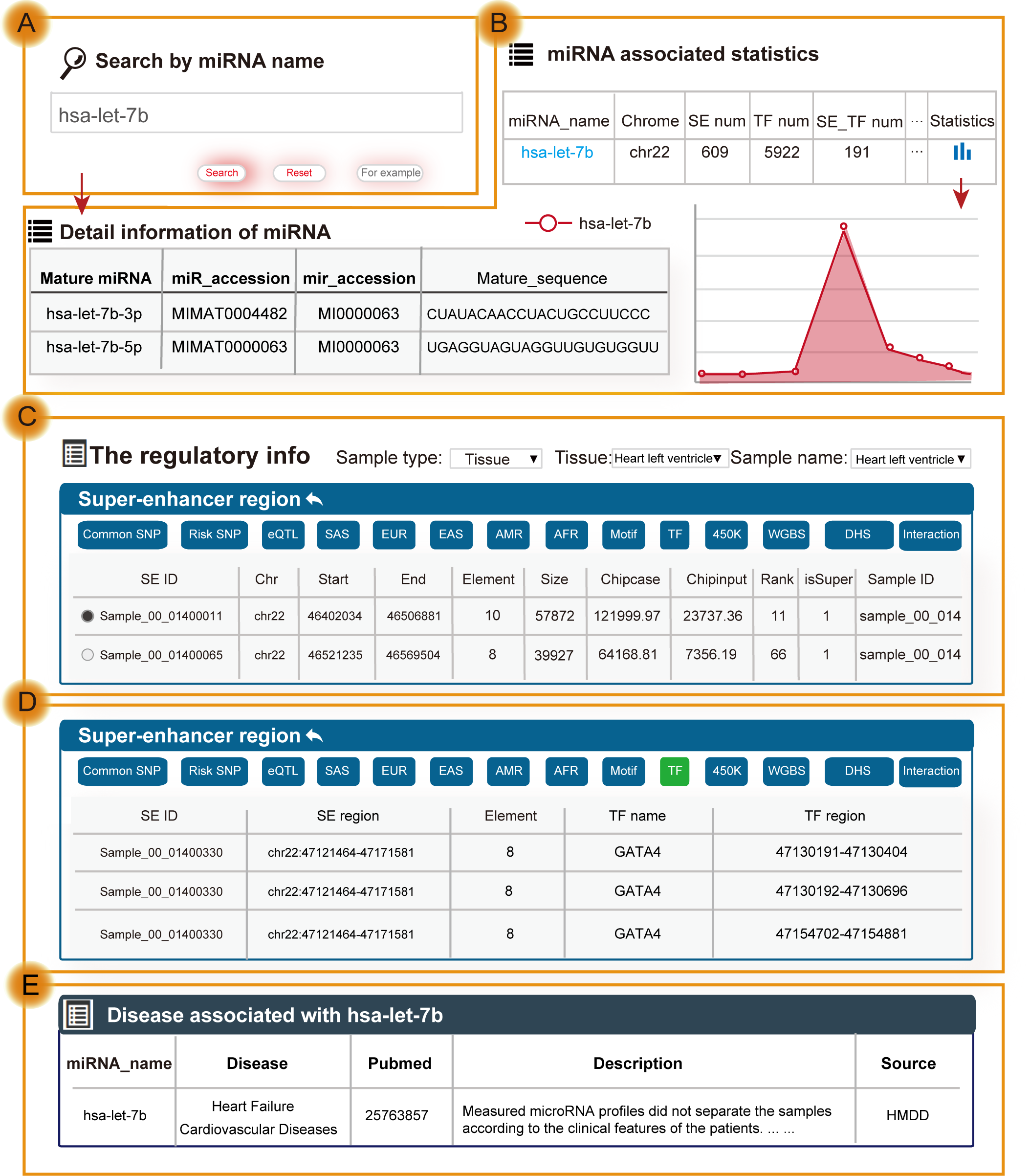

Supplement: Supplementary file 6 [file Image1.TIF]
